# Supplementary material for: Circadian Rhythm and Nitrogen Metabolism Participate in the Response of Boron Deficiency in the Root of Brassica napus
Source: Int J Mol Sci. 2024 Jul 30;25(15):8319. doi: 10.3390/ijms25158319 (PMC11313459; doi:10.3390/ijms25158319)
Supplement: Supplementary file 1 [file ijms-25-08319-s001.zip › ijms-3124879-supplementary.pdf]

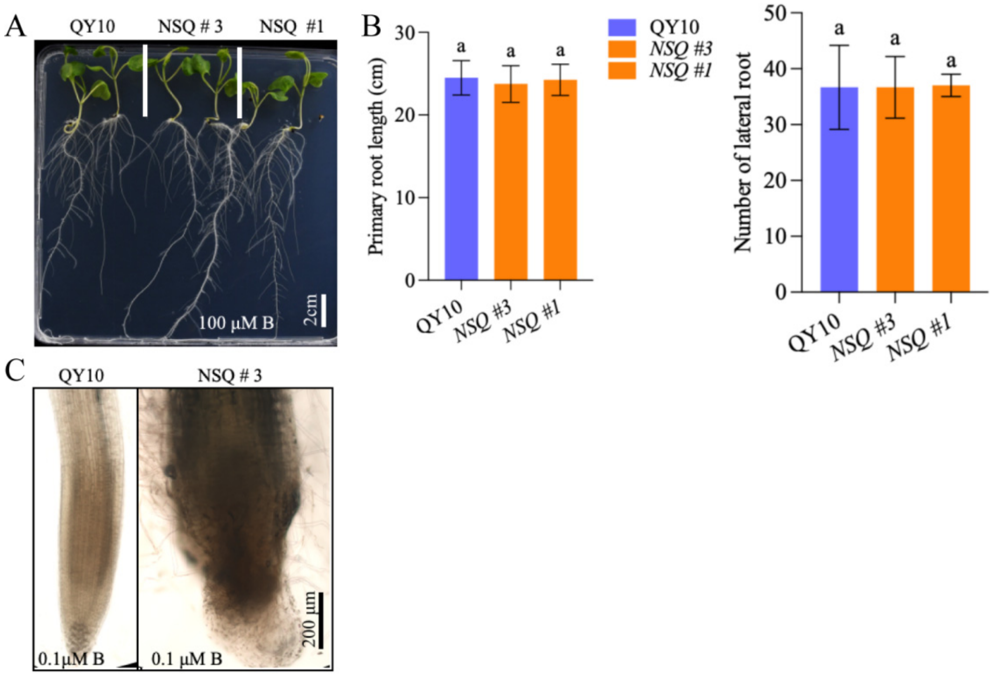

Supplementary Figure S1. Boron deficiency inhibits root growth of NSQ (BnaA3NIP5;1 RNAi lines) cultured on agar plate. The phenotype (**A**) and statistics (**B**) of primary root length and lateral root number of QY10 and NSQ lines cultured on agar plate for 10 days under 100 μM B, ($n \geq 3$). Values represent means ± SD. letters indicate significant differences between different treatments: Duncan's test (P<0.05). (**C**) The microstructure of the root tips of QY10 and NSQ #3 cultured on plate for 10 days under 0.1 μM B.

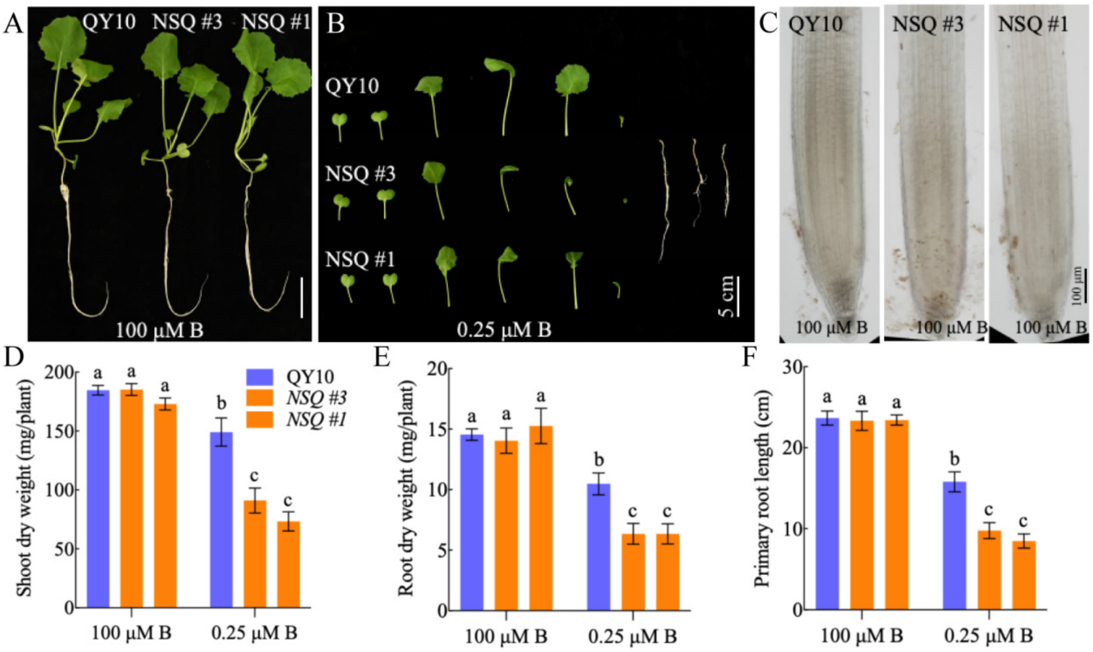

Supplementary Figure S2. Boron deficiency inhibits root growth of NSQ (BnaA3NIP5;1 RNAi lines) cultured in nutrient solution. The phenotype (**A,B**) and statistics of shoot fresh weight (**D**), root fresh weight (**E**) and primary root length (**F**) of QY10 and NSQ lines cultured in nutrient solution for 14 days under 100 μM B and 0.25 μM B; ($n \geq 3$). Values represent means ± SD. letters indicate significant differences between different treatments: Duncan's test ($P<0.05$). (**C**) The microstructure of the root tips of QY10 and NSQ lines in (**A**).

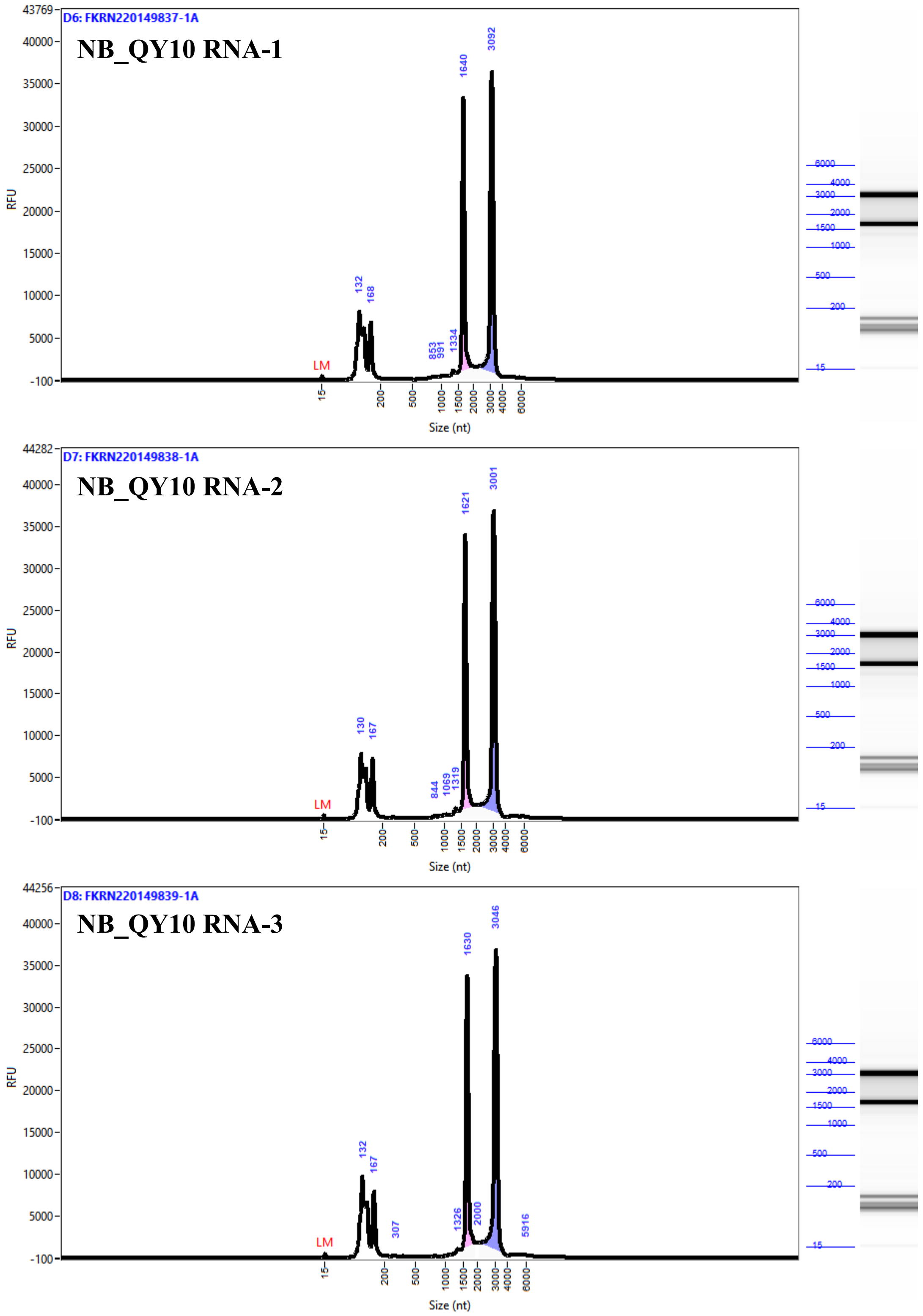

Supplementary Figure S3. Results of NB_QY10 RNA sample integrity testing. The results of RNA sample integrity testing using Agilent 5400 fragment analyzer system. Horizontal axis: size (nt) represents the distribution of fragments in the capillary sample where the reaction is located. The vertical axis (RFU) value refers to the

real-time fluorescence signal intensity of the sample during capillary separation. The higher the RFU value, the higher the sample concentration. Low Marker (LM): Refers to the reference material (non sample fragments) used for sample analysis, used to calibrate the fragment size and concentration of the sample. 25S is within the range of 3000−4000 nt, and 18S is within the range of 1500−2000 nt. On the right side are simulated agarose gels diagrams and corresponding size value annotations.

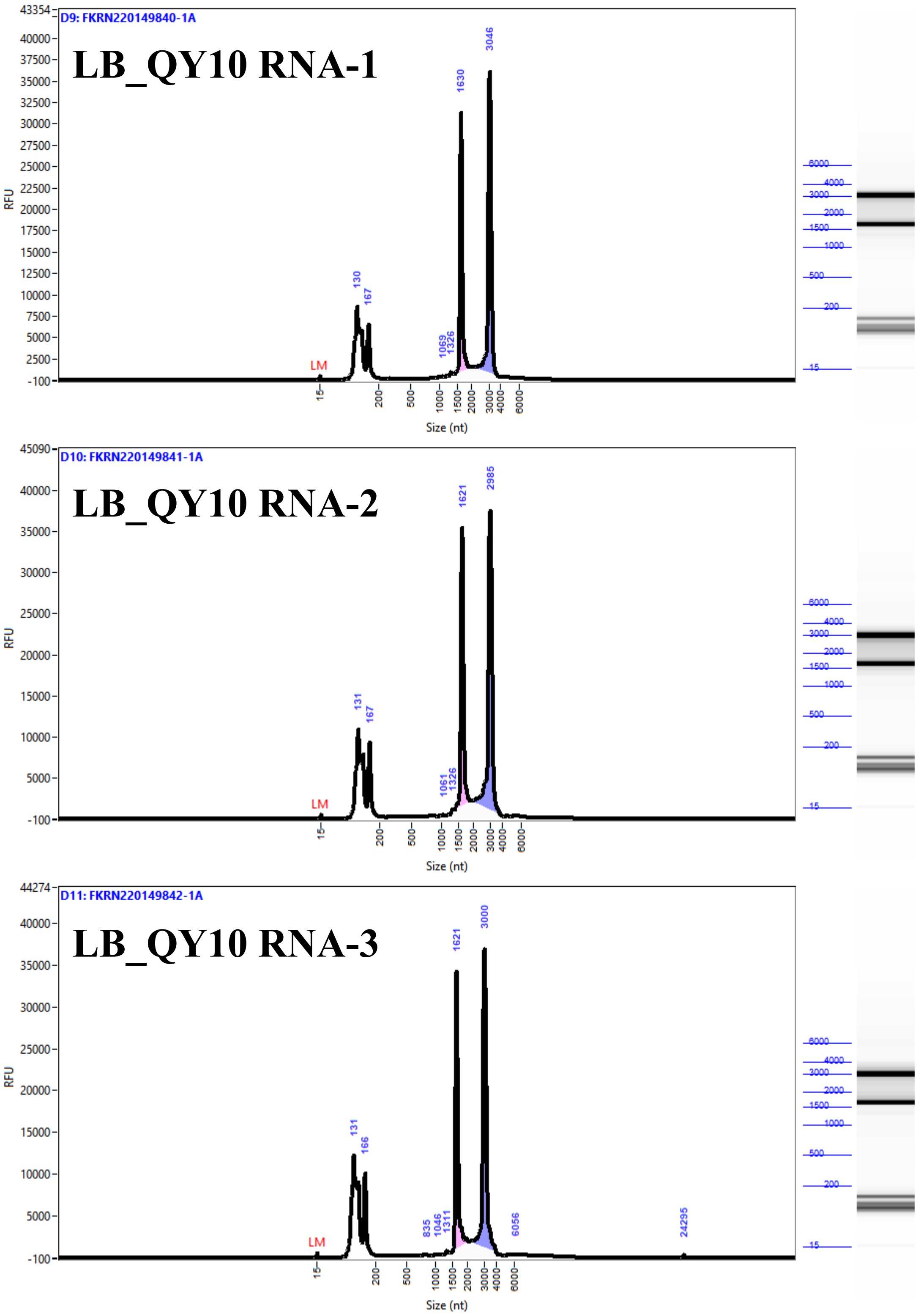

Supplementary Figure S4. Results of LB_QY10 RNA sample integrity testing.

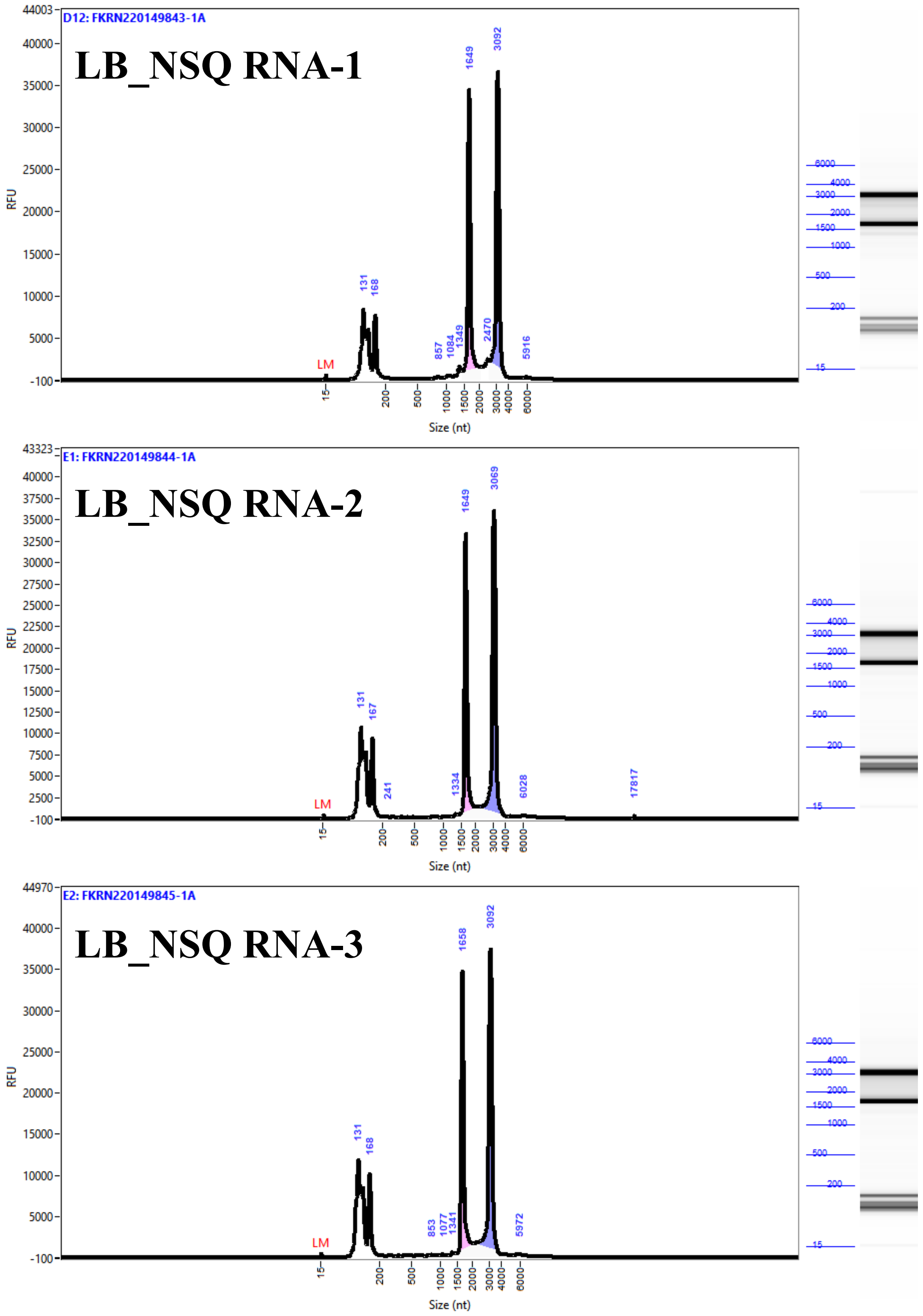

Supplementary Figure S5. Results of LB_NSQ RNA sample integrity testing.

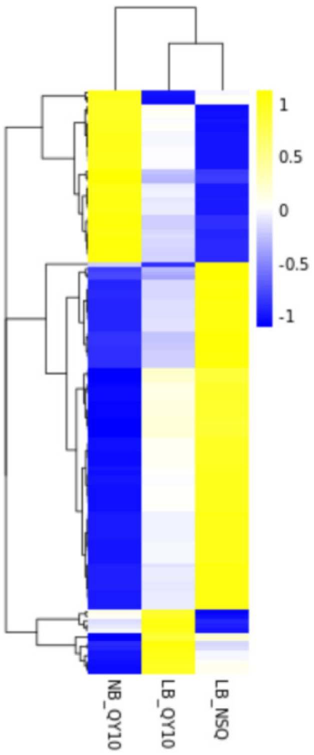

Supplementary Figure S6. Cluster analysis of DEG among NB_QY10, LB_QY10 and LB_NSQ. The horizontal axis represents the group name, and the vertical axis represents the normalized values of gene FPKM values by row after cluster analysis. From blue to yellow, the expression level is increasing.

A

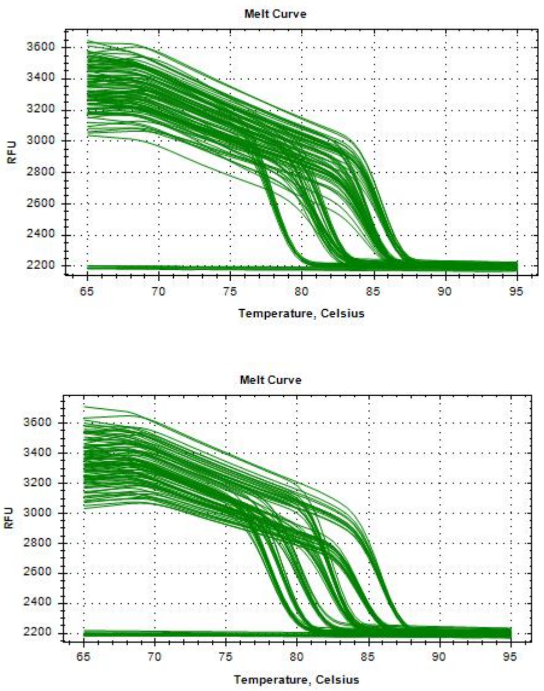

B

Supplementary Figure S7 panels A and B are in the image above.

Supplementary Figure S7. The melt curve for the selected genes in Figure 3 is presented in Panel A (first 8 genes), while Panel B includes data for the remaining genes (9−10 genes), including some not discussed in this article.

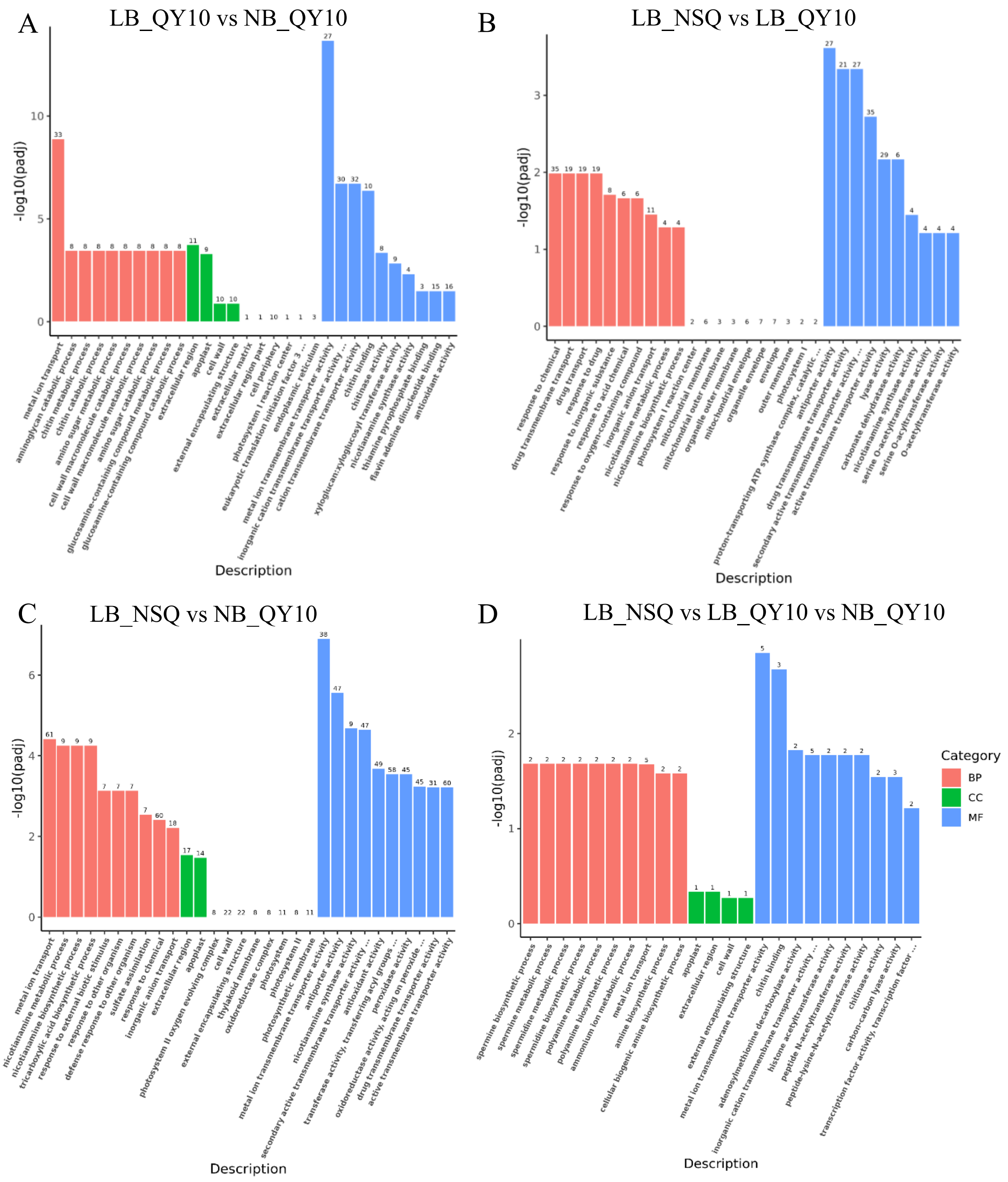

Supplementary Figure S8. GO functional enrichment analysis of DEGs in different groups. (**A**) GO terms analysis of DEGs in LB_QY10 vs NB_QY10. (**B**) GO terms analysis of DEGs in LB_NSQ vs LB_QY10. (**C**) GO terms analysis of DEGs in LB_NSQ vs NB_QY10. (**D**) GO terms analysis of DEGs in LB_NSQ vs LB_QY10 vs NB_QY10. The figures show the top10 terms (Less than 10 are all showed). The horizontal axis is the term description, and the vertical axis represents the significance level. The higher the value, the more significant it is. The number on the column

represents the number of genes enriched in this pathway. Red, green, and blue represent the three GO subclasses of BP (biological process), CC (Cellular component), and MF (Molecular function) respectively.

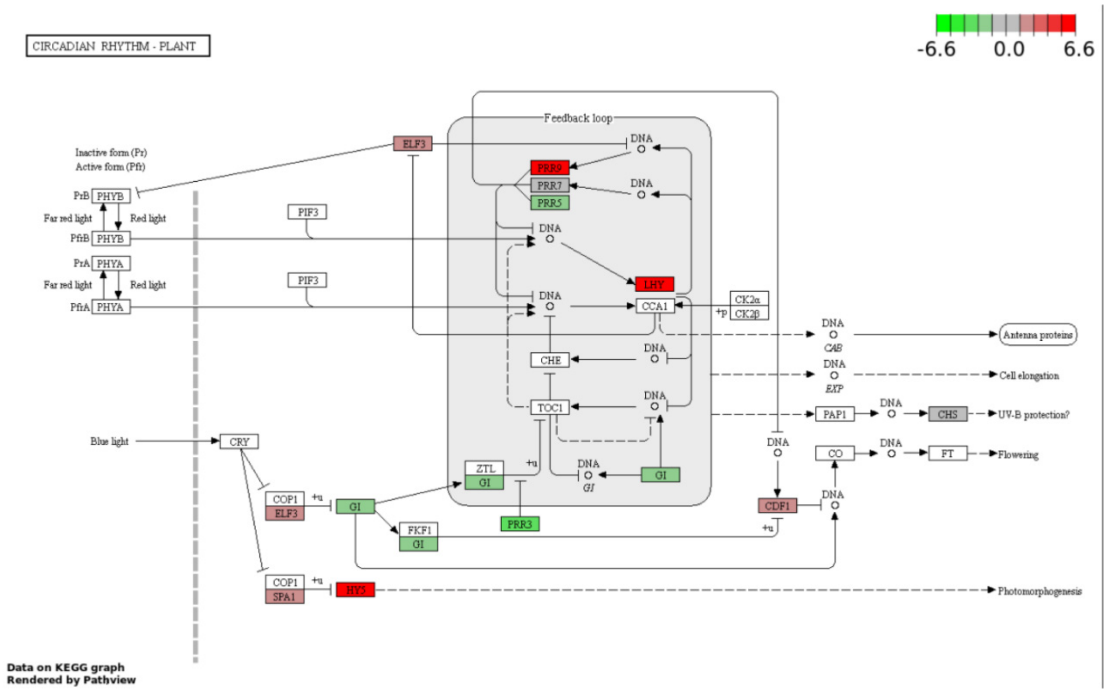

Supplementary Figure S9. Circadian rhythm pathway. Arrows represent regulatory processes, the boxes represent genes, red represents up-regulation or genes, green represents down-regulation of genes.

Table S1 RNA Sample quality inspection report.

Name	Number	Concentration (ng/ul)	Volume (ul)	Total (ug)	Value of integrity	Test conclusion
**+B QY10-1**	FKRN220149837-1A	1407.0	46.0	64.7	9.20	Pass
**+B QY10-2**	FKRN220149838-1A	1008.0	46.0	46.4	9.20	Pass
**+B QY10-3**	FKRN220149839-1A	1282.0	46.0	59.0	9.10	Pass
**−B QY10-1**	FKRN220149840-1A	1516.0	46.0	69.7	9.20	Pass
**−B QY10-2**	FKRN220149841-1A	1850.0	46.0	85.10	9.10	Pass
**−B QY10-3**	FKRN220149842-1A	1689.0	44.0	74.30	8.90	Pass
**−B NSQ-1**	FKRN220149843-1A	1598.0	43.0	68.7	9.10	Pass
**−B NSQ-2**	FKRN220149844-1A	1906.0	43.0	81.9	9.00	Pass
**−B NSQ-3**	FKRN220149845-1A	2022.0	43.0	86.9	8.90	Pass

Name : Sample Name;

Number: The sample's identification number in the sequencing company;

Value of integrity: The evaluation of RNA sample integrity, with higher values indicating better integrity;

Test conclusion: The conclusion on whether the RNA sample can be used for library construction. (Pass: The sample quality meets the quality requirements for library sequencing, and the total amount meets the requirement for one or more library constructions.)

Table S2. A summary of the basic Q.infectoria sequence statistics.

**Sample**	**Total_reads**	**Total_map**	**Unique_map**	**Multi_map**
**NB_QY10_1**	44895188	42196911(93.99%)	40221782(89.59%)	1975129(4.4%)
**NB_QY10_2**	45077376	42401506(94.06%)	40423838(89.68%)	1977668(4.39%)
**NB_QY10_3**	41169272	38668334(93.93%)	36852046(89.51%)	1816288(4.41%)
**LB_QY10_1**	41819720	39304764(93.99%)	37468393(89.6%)	1836371(4.39%)
**LB_QY10_2**	41723602	39304459(94.2%)	37467058(89.8%)	1837401(4.4%)
**LB_QY10_3**	46781234	44000952(94.06%)	41890702(89.55%)	2110250(4.51%)
**LB_NSQ_1**	45171096	42485984(94.06%)	40529861(89.73%)	1956123(4.33%)
**LB_NSQ_2**	40799492	38314109(93.91%)	36554257(89.59%)	1759852(4.31%)
**LB_NSQ_3**	44730100	42030066(93.96%)	40031397(89.5%)	1998669(4.47%)

Sample: Sample name;

Total_reads: The number of clean reads of sequencing data after quality control;

Total_map: The number and percentage of reads aligned to the genome;

Unique_map: Number and percentage of reads aligned to the unique position of the reference genome (used for subsequent quantitative data analysis reads);

Multi_map: The number and percentage of reads aligned to multiple positions in the reference genome.

Table S3. Identification and annotation of circadian rhythm pathway genes, and their homologous genes and homology in *Arabidopsis*.

Gene ID	NB_QY	LB_QY	LB_NSQ	Subject_name	Araport_id	Identity
BnaA10G0008600ZS	0.41	10.03	29.16	LHY	AT1G01060	0.82
BnaC05G0010100ZS	0.33	7.22	21.76	LHY	AT1G01060	0.86
BnaC03G0001300ZS	0.21	2.77	4.53	LHY	AT1G01060	0.94
Bnascaffold0415G0000300ZS	0.02	1.02	1.93	LHY	AT1G01060	0.93
BnaA07G0053300ZS	0.03	0.18	0.38	LHY	AT1G01060	0.93
BnaA03G0350200ZS	1.01	3.59	3.79	HYH	AT3G17609	0.89
BnaA01G0333200ZS	4.29	12.41	14.77	HYH	AT3G17609	0.86
BnaC01G0412000ZS	6.31	18.49	23.53	HYH	AT3G17609	0.85
BnaC08G0423900ZS	4.33	4.68	9.38	ELF3	AT2G25930	0.84
BnaA10G0237000ZS	20.00	46.18	47.46	HY5	AT5G11260	0.88
BnaA03G0224200ZS	2.12	5.37	5.80	SPA1	AT2G46340	0.89
BnaC02G0468600ZS	5.14	6.03	10.84	TT4	AT5G13930	0.87
BnaC09G0435100ZS	6.89	5.66	2.59	PRR3	AT5G60100	0.85
BnaA10G0154500ZS	4.21	3.27	1.32	PRR3	AT5G60100	0.88
BnaA09G0458700ZS	7.66	6.23	1.46	GI	AT1G22770	0.89
BnaC05G0198400ZS	5.83	6.26	1.52	GI	AT1G22770	0.89
BnaA04G0295200ZS	0.29	3.71	1.14	PRR9	AT2G46790	0.87
BnaA05G0011400ZS	0.00	1.08	0.82	PRR9	AT2G46790	0.87
BnaA02G0009400ZS	6.62	7.46	2.93	PRR7	AT5G02810	0.88
BnaC09G0614800ZS	12.01	19.48	6.54	PRR7	AT5G02810	0.86
BnaA03G0224100ZS	3.16	7.79	7.43	SPA1	AT2G46340	0.91
BnaA04G0172100ZS	2.63	2.39	5.56	ELF3	AT2G25930	0.83
BnaC02G0532300ZS	0.28	1.98	1.04	CDF1	AT5G62430	0.88
BnaC09G0057600ZS	0.46	0.43	0.10	PRR5	AT5G24470	0.88
BnaA06G0329000ZS	3.44	2.93	6.57	PRR5	AT5G24470	0.85
BnaC07G0364800ZS	5.91	4.47	9.28	PRR5	AT5G24470	0.88
BnaC07G0381000ZS	20.35	4.86	10.98	TOC1	AT5G61380	0.88
BnaA03G0408400ZS	7.77	2.27	5.01	TOC1	AT5G61380	0.88
BnaC09G0066100ZS	2.60	0.28	1.32	TOC1	AT5G61380	0.92
BnaC04G0214400ZS	7.55	1.34	5.27	COR27	AT5G42900	0.88
BnaA08G0138800ZS	3.22	0.27	2.72	COR28	AT4G33980	0.83
BnaA01G0041400ZS	11.50	2.25	6.62	COR28	AT4G33980	0.83

High

Low

Table S4. Identification and annotation of nitrogen metabolism pathway genes, and their homologous genes and homology in *Arabidopsis*.

Gene ID	NB_QY	LB_QY	LB_NSQ	Subject_name	Araport_id	Identity
BnaC05G0417000ZS	2.69	3.85	5.85	CYN	AT3G23490	0.93
BnaA07G0076900ZS	30.15	43.74	64.51	CYN	AT3G23490	0.88
novel.2046	0.79	2.26	2.48	CYN	AT3G23490	0.82
BnaA02G0068000ZS	0.02	0.21	0.50	NIT2	AT3G44300	0.84
BnaA02G0025800ZS	165.89	135.18	373.06	GDH2	AT5G07440	0.91
BnaA03G0027700ZS	10.33	8.37	46.36	GDH2	AT5G07440	0.89
BnaA08G0140800ZS	7.64	6.76	26.66	BCA5	AT4G33580	0.88
BnaC05G0208300ZS	0.52	0.47	1.97	BCA3	AT1G23730	0.91
novel.2217	2.05	1.52	3.38	NIT2	AT3G44300	0.84
BnaC05G0559700ZS	0.45	0.15	0.63	CA1	AT3G01500	0.92
BnaC06G0258800ZS	10.74	4.18	2.01	NIA1	AT1G77760	0.87
BnaA07G0237900ZS	9.94	4.60	2.20	NIA1	AT1G77760	0.87
BnaA02G0228500ZS	3.05	1.23	0.71	NIA1	AT1G77760	0.87
BnaC02G0204200ZS	269.34	218.70	103.34	GLN1;2	AT1G66200	0.92
BnaC07G0172200ZS	11.76	7.59	3.30	BCA3	AT1G23730	0.90
BnaC06G0369600ZS	19.01	10.32	3.41	BCA4	AT1G70410	0.88
BnaA07G0317600ZS	11.38	5.14	3.42	BCA4	AT1G70410	0.92
BnaC08G0033200ZS	9.76	8.55	2.18	NRT2:1	AT1G08090	0.89
BnaC05G0059500ZS	0.82	0.77	0.05	NRT2:1	AT1G08090	0.91
BnaC08G0532700ZS	0.51	0.35	0.01	NRT2:1	AT1G08090	0.89
BnaC08G0532800ZS	11.19	9.37	0.67	NRT2:1	AT1G08090	0.89
BnaA06G0047600ZS	12.15	9.52	1.31	NRT2:1	AT1G08090	0.90
BnaC05G0059600ZS	8.19	4.98	0.86	NRT2:1	AT1G08090	0.90
BnaC08G0033300ZS	11.77	9.40	2.20	NRT2:1	AT1G08090	0.88
BnaA09G0667800ZS	15.28	12.52	0.68	NRT2:1	AT1G08090	0.90
BnaA02G0096600ZS	2.49	0.42	0.18	NRT2.3	AT5G60780	0.89
BnaA08G0276500ZS	2.64	1.74	0.43	NRT2.5	AT1G12940	0.87
BnaC01G0301600ZS	5.83	2.12	0.71	NRT2.6	AT3G45060	0.89
BnaA01G0234200ZS	2.46	0.71	0.13	NRT2.6	AT3G45060	0.89
BnaA02G0187600ZS	67.65	69.85	18.28	BCA4	AT1G70410	0.88
BnaA06G0047500ZS	28.74	35.00	1.74	NRT2:1	AT1G08090	0.90
BnaA08G0300800ZS	9.83	11.82	2.34	NRT2:1	AT1G08090	0.88
BnaA06G0190300ZS	0.76	4.44	2.06	NIT2	AT3G44300	0.83
BnaA02G0065000ZS	308.24	430.69	192.43	GLN1;4	AT5G16570	0.92

High

Low
